# Adiposity covaries with signatures of asymmetric feedback learning during adaptive decisions

**DOI:** 10.1093/scan/nsaa088

**Published:** 2020-06-29

**Authors:** Timothy Verstynen, Kyle Dunovan, Catherine Walsh, Chieh-Hsin Kuan, Stephen B Manuck, Peter J Gianaros

**Affiliations:** Department of Psychology, Carnegie Mellon University, Pittsburgh, PA 15213, USA; Carnegie Mellon Neuroscience Institute, Carnegie Mellon University, Pittsburgh, PA 15213, USA; Center for the Neural Basis of Cognition, Carnegie Mellon University, Pittsburgh, PA 15213, USA; Department of Psychology, Carnegie Mellon University, Pittsburgh, PA 15213, USA; Center for the Neural Basis of Cognition, Carnegie Mellon University, Pittsburgh, PA 15213, USA; Department of Psychology, University of Pittsburgh, Pittsburgh, PA 15260, USA; Center for the Neural Basis of Cognition, University of Pittsburgh, Pittsburgh, PA 15260, USA; Department of Psychology, University of Pittsburgh, Pittsburgh, PA 15260, USA; Center for the Neural Basis of Cognition, University of Pittsburgh, Pittsburgh, PA 15260, USA; Department of Psychology, University of Pittsburgh, Pittsburgh, PA 15260, USA; Department of Psychology, University of Pittsburgh, Pittsburgh, PA 15260, USA; Center for the Neural Basis of Cognition, University of Pittsburgh, Pittsburgh, PA 15260, USA

**Keywords:** adiposity, decision-making, learning, obesity, basal ganglia

## Abstract

Unhealthy weight gain relates, in part, to how people make decisions based on prior experience. Here we conducted *post hoc* analysis on an archival data set to evaluate whether individual differences in adiposity, an anthropometric construct encompassing a spectrum of body types, from lean to obese, associate with signatures of asymmetric feedback learning during value-based decision-making. In a sample of neurologically healthy adults (*N* = 433), ventral striatal responses to rewards, measured using fMRI, were not directly associated with adiposity, but rather moderated its relationship with feedback-driven learning in the Iowa gambling task, tested outside the scanner. Using a biologically inspired model of basal ganglia-dependent decision processes, we found this moderating effect of reward reactivity to be explained by an asymmetrical use of feedback to drive learning; that is, with more plasticity for gains than for losses, stronger reward reactivity leads to decisions that minimize exploration for maximizing long-term outcomes. Follow-up analysis confirmed that individual differences in adiposity correlated with signatures of asymmetric use of feedback cues during learning, suggesting that reward reactivity may especially relate to adiposity, and possibly obesity risk, when gains impact future decisions more than losses.

## Introduction

The rates of obesity, defined as having a body mass index (BMI) of 30 kg/m^2^ or higher, has increased over the last two decades in the USA such that over a third of the adult population is considered obese and another third is overweight (25 < BMI < 30; Ogden *et al.,* 2010, 2014; Flegal *et al.,* 2016; Hales *et al.,* 2018), putting substantial strain on the US healthcare system for treating obesity-related illnesses (Cawley and Meyerhoefer, 2012). The speed of this population-level change in unhealthy weight gain suggests that the so-called ‘obesity epidemic’ may be driven, at least in part, by behavioral responses to altered environmental conditions (e.g. altered eating habits and activity patterns), rather than inherent changes in peripheral physiology (e.g. metabolic function). Based on the latter premise, how people make decisions may thus explain some of the variation across individuals in unhealthy weight gain.

Indeed, obesity has been associated with cognitive efficiency in a number of domains (for review, see Farruggia and Small, 2019), including executive control ability (Davis *et al.,* 2004; Nederkoorn *et al.,* 2006; Pignatti *et al.,* 2006; Gunstad *et al.,* 2007; Weller *et al.,* 2008; Duchesne *et al.,* 2010; Mobbs *et al.,* 2011; Hendrick *et al.,* 2012), mental flexibility (Cserjési *et al.,* 2007, 2009; Boeka and Lokken, 2008) and learning and memory (Duchesne and Ratelle, 2010) (for review, see Fitzpatrick *et al*., 2013). These cognitive alterations often correspond to obesity-related variability in cortico-basal ganglia pathways that support decision-making and reward processing. For example, obesity is associated with reduced D2-receptor binding (Volkow *et al.,* 2008b; de Weijer *et al.,* 2011), altered reactivity to food cues in ventral and dorsal striatal regions (Stice *et al.,* 2008, 2010; Burger and Stice, 2012), modified corticostriatal connectivity (García-García *et al.,* 2012; Marqués-Iturria *et al.,* 2015), and individual differences in genetic markers of dopamine availability (Noble *et al.,* 1994; Nisoli *et al.,* 2007; Stice *et al.,* 2008). These observations have led to the hypothesis that risk for obesity may be engendered by a cluster of neurobiological phenotypes associated with addiction, mediated by hypersensitive mesolimbic dopaminergic pathway (Volkow *et al.*, 2008a, 2012; Tomasi and Volkow, 2013).

Dopamine, however, has a complex influence on the cortico-basal ganglia networks that guide value-based decisions. A key architectural feature of these networks is the dueling influence of the direct (behavior promoting) and indirect (behavior suppressing) striatal pathways (Alexander *et al.,* 1986). Theoretical models have proposed that decisions are encoded as a dynamic competition between these two pathways (Dunovan *et al.,* 2015; Dunovan and Verstynen, 2016; Mikhael and Bogacz, 2016; Bariselli *et al.,* 2019), with the strength of evidence for a given action computed as the likelihood ratio of a hypothesis to ‘execute’ (direct pathway) *vs* a hypothesis to ‘suppress’ (indirect pathway). During learning, phasic dopamine responses, thought to reflect reward prediction errors (Schultz *et al.,* 1997), have opposing influences on the direct and indirect pathways, depending on the action that they represent and the nature of the feedback signal (i.e. gain or loss) (Bogacz and Larsen, 2011; Gurney *et al.,* 2015; Vich *et al.,* 2020). Specifically, if direct pathway neurons, with D1 receptors, are eligible for plasticity when a phasic dopamine response occurs (i.e. their firing contributed to the selected action), the likelihood of the actions encoded by those neurons increases. If, however, indirect pathway neurons, with D2 receptors, are eligible for plasticity during a phasic dopamine response, then the likelihood of actions encoded by those neurons decreases.

Thus how reward feedback impacts future decisions depends, in large part, on the relative sensitivity of these two competing striatal pathways to phasic dopamine signals (Cools *et al.,* 2009; Frank and Hutchison, 2009). Indeed, recent computational theories (Morimura *et al.,* 2010; Bellemare *et al.,* 2017; Dabney *et al.,* 2018), supported by emerging neurophysiological evidence (Dabney *et al.,* 2020), suggest that the value of potential actions are represented as a distribution, with different populations of striatal pathways reflecting degrees of optimism or skepticism for the value of any given action. This distributional form of reinforcement learning is thought to arise in basal ganglia pathways due to asymmetries in the relative plasticity of coupled direct and indirect pathways (Dabney *et al.,* 2020), with ‘skeptics’ (i.e. pathways representing low value for an action) having higher sensitivity to errors than successes and vice versa for ‘optimist’ representations. The overall distribution of ‘skeptics’ and ‘optimists’ for any given action determines how an agent uses feedback to guide future decisions. This means that differences in relative learning from positive (i.e. increasing D1 synaptic efficacy of direct pathways) and negative (i.e. increasing D2 synaptic efficacy of indirect pathways) reward feedback might explain individual differences in value-based decisions that, in turn, associate with decision-mediated health outcomes.

These insights from the computational neurobiology of adaptative decision-making suggest that if variation in obesity is driven by differences in value-based decision-making, then body type should covary with both reward reactivity and asymmetries in how gains and losses impact future decisions. Here we leveraged archival analysis on a previously published data set to evaluate how individual differences in adiposity—a general anthropometric construct that encompasses facets of body habitus, fatness, and obesity—associates with ventral striatal reward reactivity, measured using fMRI responses to monetary wins *vs* losses, and feedback-driven learning using a popular reinforcement learning task. In subsequent computational modeling, we used a biologically inspired model of basal ganglia-dependent decision processes to identify a signature of asymmetric use of feedback (gains *vs* losses) that could be used to confirm whether individual differences in adiposity associate with asymmetries in feedback learning that are moderated by reward reactivity.

## Materials and methods

### Participants

Data for the present study were derived from the University of Pittsburgh Adult Health and Behavior project, Phase II (AHAB-II). AHAB-II is a registry of behavioral and biological measurements in a sample of community dwelling adults, aged 30–54-years old and recruited via mass-mail solicitation from communities of southwestern Pennsylvania, USA. General inclusion and exclusion criteria for recruitment are described elsewhere (Gianaros *et al.,* 2014; Marsland *et al.,* 2015). Informed consent was obtained in accordance with approved protocol guidelines of the University of Pittsburgh Internal Review Board. Of the full sample of 490 individuals, only individuals who were tested on the Iowa gambling task (IGT), had reliable imaging data to assess ventral striatum (VS) reactivity (see below), and had all relevant anthropometric measures were included for analysis. This resulted in a final analytical sample of 433 adults. Demographics of the sample are described in Table 1. All archival analysis procedures were approved by both the Carnegie Mellon University and University of Pittsburgh Internal Review Boards.

**Table 1:** Demographics and key analysis variables. See main text for descriptions of variables

	Mean	STD	Range
Age	42.76	7.35	(30, 54)
Female	52.19%		
Caucasian	82.91%		
Education (years)	16.96	2.85	(9, 24)
Adiposity	0.003	1.00	(−2.26, 2.72)
BMI	26.73	4.96	(17.50, 45.10)
Waist circumference (in)	35.54	5.48	(24.50, 52.00)
Payoff (P)	16.16	29.97	(−92, 96)
Sensitivity (Q)	31.55	25.21	(−46, 86)
VS reactivity	0.11	0.17	(−0.54, 0.85)

### Adiposity score

**Fig. 1:**
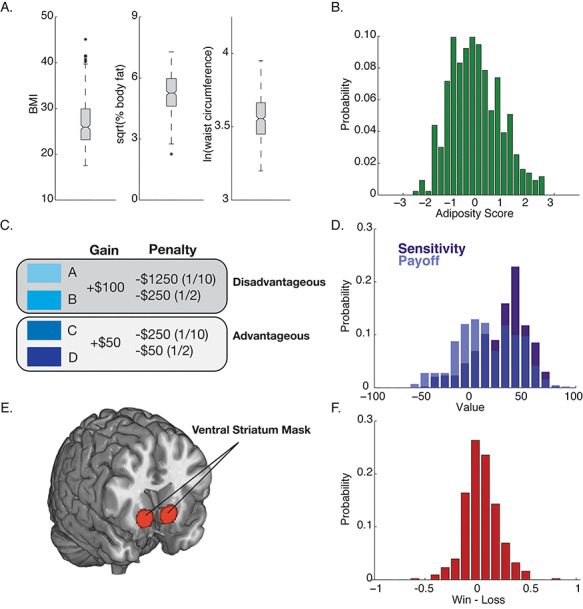
Anthropometric, behavioral and neural measures. (A) Whisker plots of variables used in the calculation of the adiposity score. The box range shows the 25th and 75th confidence intervals. Asterisks show outliers outside the 95th percent confidence intervals. (B) Distribution of adiposity scores across participants (see Methods). (C) Outline of the IGT. Participants select a card from one of four decks with pre-determined gain/loss outcomes. Two decks, A and B, provide large immediate gains, but lead to long-term losses. Two decks, C and D, provide small immediate gains, but overall lead to long-term payoffs. (D) Distributions of payoff and sensitivity scores (see Methods) across participants. (E) Region of interest masks used in the cards task to measure reward reactivity in the VS. (F) Distribution of VS reward reactivity scores across participants.

Of the original sample of 490 participants, four participants were missing body fat values. Rather than rely on individual proxy measures of obesity and adiposity, that each have their own unique limitations (e.g. BMI is confounded by overall body size), we created a composite variable that captures common variance across multiple measures of body type. A composite adiposity score was constructed from the remaining 483 participants using three indicators: percentage of total body weight that is fat (FAT), BMI and waist circumference (WST; log transformed) (Figure 1A, Table 1). In order to meet the assumptions of principal component analysis (PCA), FAT was square root transformed and WST was natural log-transformed indicators to achieve normality. No outstanding multivariate outliers were identified using the Mahalanobis distance statistic. Box-Cox tests and collinearity statistics (VIFs) were less than 10, suggesting the absence of multi-collinearity. Indicators were sufficiently correlated, having a Kaiser–Meyer–Olkin measure of 0.538 and Bartlett’s test of sphericity chi-square of 922.791. A single-factor, adiposity composite score was extracted using PCA-regression, explaining 76.89% of the shared variance across variables (Figure 1B). Standardized factor loadings (or beta weights) for WST, BMI and FAT were 0.880, 0.962 and 0.779, respectively. Normality of the extracted factor scores was examined by the Kolmogorov–Smirnov (K–S) test, with scores being normally distributed (K–S value = 0.037). Three outliers were identified with values greater than three standard deviation from the mean and removed from analysis. Analysis was performed using SPSS v21.

### Iowa gambling task

To measure the efficiency of adaptive decision-making, we used a computerized version of the IGT (Figure 1C; Bechara, 2007), that has a high reliability and construct validity across both normal and clinical populations (Buelow and Suhr, 2009) (though see also Lin *et al.,* 2013). The computerized IGT consists of four decks of 60 cards each, where choosing decks A and B results in short-term gains but long-term losses, and decks C and D the reciprocal. Decks A and C result in higher frequency losses and decks B and D result in lower frequency losses. Participants start with a virtual amount of $2000 and are instructed that the goal is to maximize winnings and that some decks are better than others. Once a card is chosen, a message is revealed indicating that the player has won some money, lost some money or a combination. The total amount of money won or lost is then indicated by addition or subtraction from the starting amount. The order of the cards in each deck is the same for each participant. The task lasts for 100 trials although the participant is told that the game may end at any time. Payoff is calculated by subtracting the number of times a short-term payoff deck was selected from the number of times a long-term payoff deck was selected: Payoff = (C + D) – (A + B); while a participants’ sensitivity to frequency of punishment and reward is calculated as the difference between the high and low frequency loss decks: Sensitivity = (B + D) – (A + C) (Stocco *et al.,* 2009; Figure 1D).

### VS reactivity task

Ventral striatal reactivity was measured using a standard monetary gain task that has been shown to have the sensitivity to capture individual variability in reward sensitivity (Hariri *et al.,* 2006; Gianaros *et al.,* 2011; Luking *et al.,* 2017) The task was made up of a total of 45 trials, split up into 9 blocks with 5 trials each. The subject was first shown a ‘?’ in the center of a card for 3 s. This indicated that the subject would need to guess whether the following card would be lower or higher than the number 5. They indicated their choice by using a button pressing system. The index finger signaled they were selecting less than five and the middle finger signaled greater than five. The subject was then presented with a card indicating the number the computer selected. This was presented for 500 ms, followed by 500 ms of a feedback arrow depending on the correctness of the subject’s response. The green up arrow indicated a positive feedback and the red down arrow indicated negative. At the end of each trial a cross hair was then presented for 1.5 s, making the entire trial length 5.5 s. Each block was made up of five trials. There were three different conditions: win, loss and control. The win condition consisted of four out of five positive feedback responses (80% correct) with one out of five having negative feedback responses (20% incorrect), and the loss condition was the opposite. During the control blocks, the subject was presented first with an ‘x’ for 3 s. They were instructed to press their either index or middle finger in response to the presentation of the ‘x.’ They then were shown an asterisk for 500 ms and a yellow circle for 500 ms. Before the start of each block, there was a 3 s instruction shown indicating the section to follow, ‘guess number’ for the positive and negative feedback conditions and ‘press button’ for the control condition. The total task length was 350 s, including the first 6 s, which were removed to allow for magnetic equilibration.

### BOLD data acquisition and processing

The functional blood oxygenation level-dependent (BOLD) images were collected on a 3 T Trio TIM whole-body scanner (Siemens, Erlangen, Germany) using a 12-channel phased-arrayed head coil. The functional BOLD image acquisition parameters were: (FOV) = 200 × 200 mm, matrix = 64 × 64, repetition time (TR) = 2000 ms, echo time (TE) = 29 ms and flip angle (FA) = 90°. Thirty-four slices were collected each 3 mm thick with no gap in an inferior to superior direction. A total of 172 BOLD volumes were collected throughout the duration of the task. For spatial co-registration of the BOLD images, T2 weighted neuroanatomical images were acquired over 2 min 6 s by these parameters: FOV = 200 × 200 mm, matrix = 256 × 256, TR = 3000 ms, inversion time (TI) = 100 ms, TE = 11/101 ms, and FA = 150° (36 slices, 3 mm thick, no gap). A small mirror was attached to the head coil to allow the subject to see the projector placed behind them while in the scanner.

The functional BOLD images were processed using the Statistical Parametric Mapping software (SPM8; Wellcome Trust Centre for Neuroimaging, London, UK). Before analyses, BOLD images were realigned to the first image of the series by a 6-parameter rigid-body transformation, with the unwarp procedure in SPM applied to adjust for geometric distortion due to movement. Realigned images were co-registered to each participant’s T2-weighted structural image. Co-registered images were normalized by a 12-parameter nonlinear and affine transformation to the International Consortium for Brain Mapping 152 template (Montreal Neurological Institute; MNI). Normalized images were smoothed by a 6 mm full-width-at-half-maximum Gaussian kernel.

After preprocessing, linear contrast images reflecting relative BOLD signal changes (i.e. win *vs* loss, loss *vs* win and win *vs* control) were estimated for each participant. To this end, task conditions were modeled with rectangular waveforms convolved with the default SPM hemodynamic response function. Regressors were designed to model the entire win, loss or control blocks, rather than individual trials within each block. Contrast images were then generated by general linear model (GLM) estimation using an explicit brain mask and incorporating outlier weighting using the robust-weighted least-squares toolbox (Diedrichsen and Shadmehr, 2005). Before estimation, low-frequency BOLD signal noise was removed by high-pass filtering (128 s cut-off). Finally, regression vectors derived from the realignment step were included in the GLMs to account for BOLD signal variance attributable to head movement. Individual contrast images were then submitted to group-level, one-sample *t*-tests. The mean BOLD contrast parameter estimates were extracted from a predefined VS ROI (Figure 1E and F, Table 1). Voxels within the ROI exhibited condition-specific effects at FDR ≤ 0.05 and *k* ≥ 10 voxels. Bilateral (i.e. left and right VS) parameter estimates were then averaged for further analysis. The *a priori* ROI mask is available at http://bnl.pitt.edu/resources.html, and the steps taken to create it are described elsewhere (Gianaros *et al.,* 2011).

### Adaptive network model

To model value-based decisions at the implementational level, we performed simulations of the IGT using a hybridization of accumulation-to-bound (Ratcliff, 1978) and reinforcement learning models (Sutton and Barto, 1998) informed by the architecture of cortico-basal ganglia pathways (Bogacz *et al.,* 2006; Caballero and Gurney, 2010; Ratcliff and Frank, 2012; Dunovan *et al.,* 2015, 2019; Dunovan and Verstynen, 2016; Pedersen *et al.,* 2016). Here the network represents four competing decisions, one for each deck, as individual action channels that accumulate evidence toward a decision boundary (Figure 4A). The certainty of each action is reflected in the drift rate of the decision process (Dunovan *et al.,* 2015, 2019; Dunovan and Verstynen, 2016; Mikhael and Bogacz, 2016; Bariselli *et al.,* 2019; Dunovan and Verstynen, 2019) reflecting the competition between an action-promoting decision process (i.e. Go process, direct pathway), and an action-suppressing process (i.e. No Go process, indirect pathway). Inputs to each action channel follows a ‘center-surround’ topology (Mink, 1996), such that each deck is associated with only one Go pathway, but multiple No Go pathways. The differential activity of these competing processes is integrated at an output node that accumulates to a decision boundary (Dunovan *et al.,* 2015), with the first output unit to reach its decision boundary being the selected action (Dunovan *et al.,* 2019).

The decision network specifically followed the general structure of adaptive, multi-choice learning described elsewhere (Dunovan and Verstynen, 2016; Dunovan *et al.,* 2019) and the strengths and weakness of this adaptive accumulator approach have been described elsewhere (Ratcliff and Frank, 2012). Four independent action channels, reflecting each deck choice, were initiated on each trial as a competing pair of Go (G) and No Go (N) accumulators. For each action channel *j* and each trial *t*, the stepwise dynamics for the *G* and *N* accumulators was defined at each time step τ (Δτ = 1 ms) as

(1) \begin{equation*} {G}_{j,t}\left(\tau \right)={G}_{j,t}\left(\tau -\Delta \tau \right)+{\nu}_{j,t}^G\Delta \tau +{\varepsilon}_j^G\left(\tau \right) \end{equation*}

and

(2) \begin{equation*} {N}_{j,t}\left(\tau \right)={N}_{j,t}\left(\tau -\Delta \tau \right)+{\nu}_{j,t}^N\Delta \tau +{\varepsilon}_j^N\left(\tau \right) \end{equation*}

With ν^G^ and ν^N^ defining the drift rates of the G and N processes, respectively. The diffusion noise on each follows a normal distribution with variance σ^2^ = 0.01.

(3) \begin{equation*} {\varepsilon}_j^{G/N}\sim N\left(0,{\sigma}^2\right) \end{equation*}

The execution process Θ for each action channel *j* is defined as the difference between the *G* and *N* processes, such that

(4) \begin{equation*} {\Theta}_{j,t}\left(\tau \right)=\left[{G}_{j,t}\left(\tau \right)-{N}_{j,t}\left(\tau \right)\right]\cdot \cosh \left(\gamma \cdot \tau \right) \end{equation*}

The hyperbolic cosine term introduces a dynamic bias in the signal that approximates a collapsing decision boundary (Ratcliff and Frank, 2012; Dunovan *et al.,* 2015) at a rate determined by the parameter γ. Each trial simulation continues until the first deck execution process reaches the decision boundary *a*.

To get the system to learn we used feedback from gains (i.e. positive reward prediction errors; RPEs), or losses (i.e. negative RPEs) as training signals to adapt the network on a trial-by-trial basis, in a similar way as dopaminergic signals arising from the substantia nigra pars compacta influence striatal pathways (Schultz and Dickinson, 2000). Positive RPEs sensitize D1-expressing cells of the direct pathway and depress the D2-expressing cells of the indirect pathway. In contrast, negative RPEs have the opposite effect, enhancing the sensitivity the indirect pathway while depressing those of the direct pathway (Gurney *et al.,* 2015). This opposing plasticity effect means that gains reinforce the appropriately selected action over less rewarding alternatives and push the network into a more exploitative state; while losses reduce the saliency of the selected action which allows for more competition between the action channels, pushing the network into a more exploratory state (Cools *et al.,* 2009; Stauffer *et al.,* 2014; Collins and Frank, 2015; Dunovan *et al.,* 2019; Vich *et al.,* 2020) Because the drift rate of individual action decisions reflects competition between the direct and indirect pathways within an action channel (Dunovan and Verstynen, 2016; Dunovan *et al.,* 2019), we used positive and negative RPEs to modulate the relative Go and No Go drift rates of each action channel independently (Dunovan *et al.,* 2019)

As the network learns the value of each deck, it must decide what to do with this information, i.e. does it simply select the highest value deck (exploitation) or keep searching for possible better payoffs (exploration)? For this, we simulated networks with different levels of ‘greediness’ in their decision policy. At the end of each simulated trial, the network received a feedback signal, *r_t_*, reflecting the value of the card it selected. This signal was used to update the state value of each deck, *Q_j,t_*, such that

(5) \begin{equation*} {Q}_{j,t+1}={Q}_{j,t}+\lambda \left({r}_t-{Q}_{j,t}\right) .\end{equation*}

On trials with positive feedback (i.e. gain) λ = α^G^, while on trials with negative feedback (i.e. loss) λ = α^N^ (see also Collins and Frank, 2014). This state value function was then used to update the action selection probability *P*(*j*) for each deck *j* given by the softmax probability function (Sutton and Barto, 1998).

(6) \begin{equation*} P{(j)}_{t+1}=\frac{e^{\beta \cdot{Q}_{j,t+1}}}{\sum_i^4{e}^{\beta \cdot{Q}_{j=i,t+1}}} \end{equation*}

where β is the inverse temperature parameter and *Q_j,t_* is the current value estimate from equation 5*.* Greediness is therefore represented by this inverse temperature parameter (β), and the degree to which the system exploits feedback signals in order to maximize expected gain on future trials *vs* taking chances and exploring the space of decisions in order to find potential better gains. In this case, higher β values reflect a more greedy or exploitative network, while lower β values reflect a more random or exploratory network (Sutton and Barto, 1998).

Typically, *P*(*j*) is estimated for each deck and used to perform a weighted selection from the set of possible alternatives. Here, the change in *P*(*j*)*_t_* from the previous trial *P*(*j*)_*t*–1_ is calculated to obtain an estimate of the change in choice probability for each deck, *δ_j,t_*

(7) \begin{equation*} \delta{(j)}_t=P{(j)}_t-P{(j)}_{t-1} \end{equation*}

This additional step effectively converts the *Q*-value update from eq. 5 into proportional change in selection probability for each channel. This ‘choice probability’ error signal is then used to update the relative drift rates of the G and N processes in each action channel accordingly:

(8) \begin{equation*} {\nu}_{j,t}^G={\nu}_{j,t-1}^G+{\alpha}^G\cdot{\delta}_t \end{equation*}

(9) \begin{equation*} {\nu}_{j,t}^N={\nu}_{j,t-1}^N+{\alpha}^N\cdot -{\delta}_t \end{equation*}

Each network simulation followed the same trial procedures as participants in the IGT described above (100 total trials with same feedback schedules as experimental subjects). The simulation outcomes were summarized as the average results of 140 simulated agents for each value of β, α^G^ and α^N^. The following parameters were held constant in all simulations except those shown in Figure 4F: boundary height (*a* = 0.4), non-decision time (tr = 200 ms), dynamic gain (γ = 1.5) and initial drift-rates (e.g. prior to learning) for the Go (ν^G^ = 0.7) and No Go (ν^N^ = 0.4) decision variables. For each combination of α^G^ and α^N^ being compared in Figure 4F, a group of 100 agents were simulated, each with randomly sampled set of model parameters. Parameters were sampled from the following distributions (*G* = gamma, *N* = normal, *U* = uniform): a ~ *G*(0.35, 0.05), tr ~ *G*(0.2, 0.02), γ ~ *N*(0.8, 0.2), ν^G^ ~ *U*(0.7, 1.0), ν^N^ ~ *U*(0.3, 0.7), β ~ *U*(2.5, 7.0).

## Results

### Adiposity, striatal reward reactivity and decision-making

A summary of the key empirical variables is shown in Figure 1 and reported in Table 1. Relationships between all variables were evaluated using a set of bootstrapped linear regression models, with bias corrected and accelerated confidence intervals (Diciccio and Efron, 1996; Table 2). Adiposity scores were negatively associated with both payoff and sensitivity scores in the IGT, but not with VS reactivity to wins over losses. In fact, of all the pairwise factors, the only variable that significantly associated with VS reactivity was payoff score. VS reactivity and payoff score were positively associated when payoff score was used as either the predictor variable or the outcome. The positive direction of the association between payoff score and VS reactivity suggests that greater VS reactivity to reward in the cards task is linked more effective value-based decision-making in the IGT.

**Table 2:** Regression model parameters for all factors. Rows show individual response variables and columns show the predictor variables. Standard deviations of parameter fits are shown in parentheses. Logistic regression was used for all models where binary factor for gender was the outcome variable

	Adiposity	Payoff	Sensitivity	VS	Age	Gender
Adiposity		−0.005 (0.002)^**^	−0.005 (0.002)^**^	−0.106 (0.284)	0.029 (0.007)^**^	0.045 (0.099)
Payoff	−4.811 (1.388)^**^		−0.189 (0.057)^*^	21.447 (7.619)^*^	−0.337 (0.186)^***^	−10.649 (2.854)^**^
Sensitivity	−3.102 (1.143)^*^	−0.134 (0.043)^*^		2.824 (7.251)	0.264 (0.172)	10.063 (2.458)^**^
VS	−0.003 (0.008)	0.001 (0.0002)^*^	0.0001 (0.0003)		0.001 (0.001)	−0.010 (0.016)
Age	1.543 (0.327)^**^	−0.020 (0.012)^***^	0.0225 (0.014)	2.040 (1.982)		1.970 (0.708)^*^
Gender	0.011 (0.024)	−0.003 (0.001)^**^	0.004 (0.001)^**^	−0.092 (0.146)	0.0091 (0.003)^*^	

^*^ *P* < 0.01

^**^ *P* < 0.001

^***^ *P* < 0.05

**Fig. 2:**
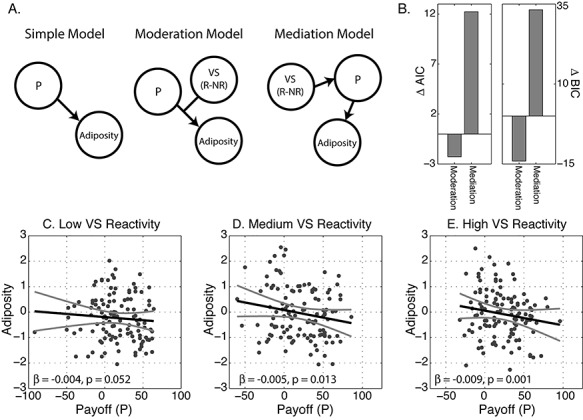
Model comparisons. (A) Depictions of the three models evaluated. See text for details. (B) Model fits using the Akaike information criterion (AIC) and Bayesian information criterion (BIC), shown as a difference between the moderation and mediation model fits and the simple model. (C–E) Adiposity-payoff associations for low, medium and high VS reward responders.

We next modeled individual differences in adiposity using three different models that were informed by the associations above. In these models, we restricted analyses to payoff scores as a measure of IGT performance because sensitivity scores did not associate with VS reactivity. As a baseline, we tested the simple model of regressing payoff score against adiposity (same as reported in Table 2). We then fit a model where VS reactivity moderated the relationship between payoff and adiposity. Finally, we fit a mediation model where VS reward reactivity indirectly associates with adiposity through payoff scores. This reflects an alternative hypothesis, where overall sensitivity to rewards solely drives value-based learning that, in turn, drives variability in adiposity. Here confidence intervals on the mediation and moderation models were estimated using bootstrapping approaches described elsewhere (Preacher and Hayes, 2008). Using two different information criterion measures, we found that the moderation model provided a substantially better fit to the data than the simple model (Figure 2B). In contrast, the mediation model did not outperform the simple model, once we controlled for model complexity (i.e. number of free parameters).

To explicate the nature of this interaction effect in the moderation model we applied a tertile split on the distribution of VS scores, and categorized subjects as having low (*N* = 143), medium (*N* = 144) or high (*N* = 146) VS reactivity. The payoff-adiposity association was then modeled separately within each group after accounting for age and sex. Figure 2C–E shows that individuals with high VS reactivity had a stronger negative correlation between payoff and adiposity scores than individuals with lower VS reactivity.

### Mechanisms of adaptive decision-making

**Fig. 3:**
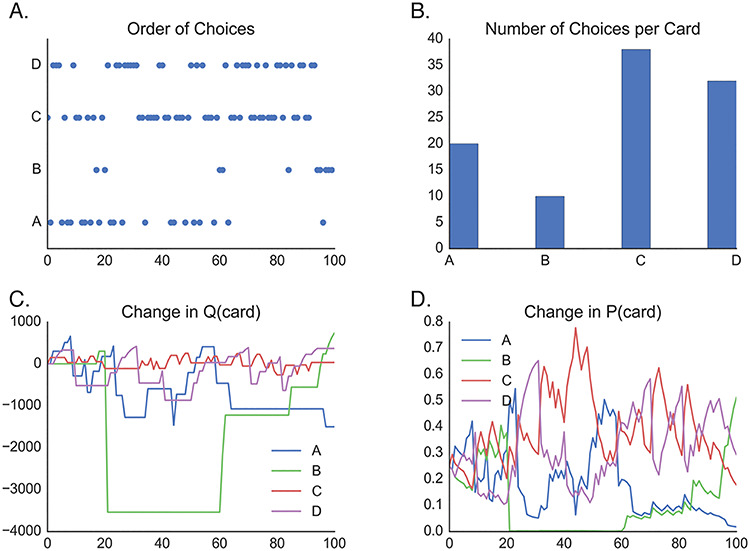
Example single network run. (A) Deck selections by the network on each trial. (B) Histogram of selections shown in (A). (C) Change in state value function (Q) on each trial based on feedback scores. (D) Change in action policy (P) for each deck option based on changes in the state value function.

**Fig. 4:**
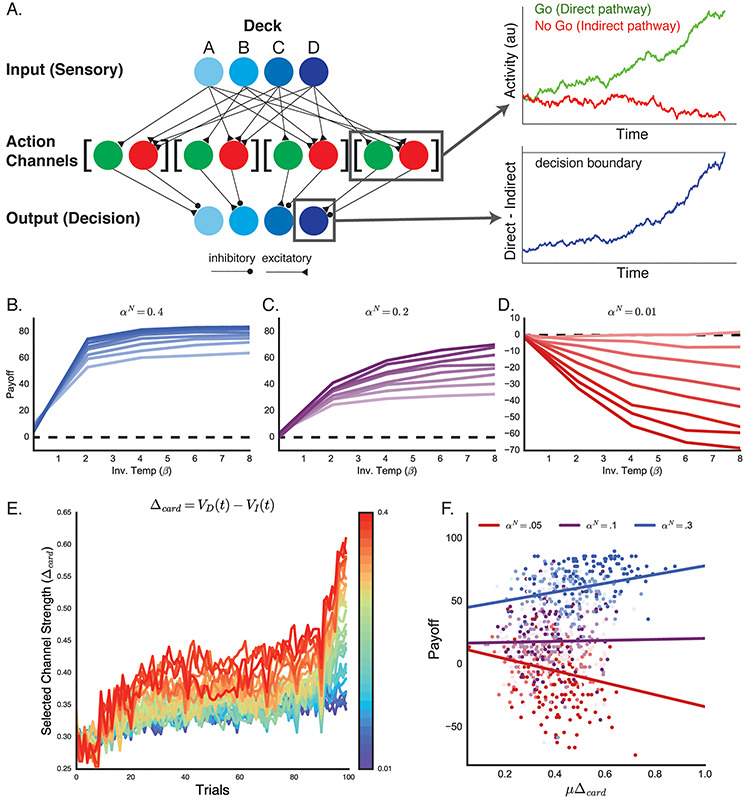
Adaptive network model. (A) Design of the adaptive Believer-Skeptic model (Dunovan and Verstynen, 2016). Each deck cue sends a drift rate to the Go pathway of a single action channel and contributes to the drift rates of No Go pathways in the remaining action channels (i.e. center-surround architecture). Within each action channel (right inset), the Go and No Go pathway activity accumulate independently and project to a single output decision node. The output decision node takes the difference of Go and No Go pathway activity, along with a dynamic bias that increases with time, and accumulates to a decision boundary. The network chooses the first output node to reach its decision boundary. See Methods for details. (B–D) Simulated payoff scores for a set of networks with a range of Go pathway learning rates (α^G^), between 0.01 (lighter lines) and 0.4 (darker lines), and softmax temperature parameter (β). Separate models were run with No Go pathway learning rates (α^N^) of 0.4 (B), 0.2 (C) and 0.01 (D). (E) Example simulations of reactivity of the network (Δ_Card_) across trials. As learning progresses, the selected pathway becomes more rewarded and reactivity increases. Warmer colors reflect network simulations with higher α^G^. (F) Relationship between network reactivity and payoff scores. Payoff scores for 100 simulated agents were regressed on trial-averaged values of the network proxy for VS reactivity (Δ_Card_) assuming different Go (α^G^) and No Go (α^N^) learning rates. Low (0.05), medium (0.1) and high (0.3) values of α^N^ are denoted by red, purple and blue, respectively. The saturation of each dot denotes the strength of α^G^, with low, medium and high values (same conventions as above) corresponding to light, medium and dark hues (each simulated agent had a β sampled from a uniform distribution between 2.5 and 7.0). As α^N^ decreases, the relationship between network reactivity and payoff score goes from a positive association to a negative association.

We simulated multiple runs of the IGT under different configurations of our cortico-basal ganglia inspired network model (see Methods). Our simulations show that the network is able to use the feedback on each trial to learn to alter the state action value for each deck (an example experimental run is shown in Figure 3). A critical question is how learning rate and decision policy sculpt the network’s decisions over time. Figure 4B–D shows the output of the network with different levels of greediness (β), learning on the Go pathway (α^G^), and learning on the No Go pathway (α^N^). In each panel α^G^ ranges from 0.01 (lighter lines) to 0.4 (darker lines). When α^N^ is high (Figure 4B and C), increasing both the greediness of the network and α^G^ improves the efficiency of the network’s decision and payoff scores increase. With a low α^N^ (Figure 4D), however, the performance of the network reverses such that greedier networks and networks with high α^G^ both tend to produce lower payoff scores. Thus, when the network has greater sensitivity to positive than to negative feedback signals, it becomes reward seeking and makes less long-term effective decisions.

### Adiposity correlates with signatures of asymmetric learning rates

The preceding analysis suggests that the general sensitivity to feedback signals will predict either more or less effective decision strategies depending on the relative efficiency of learning from gains (α^G^) relative to learning from losses (α^N^). We next examined how asymmetrical learning rates would be reflected in the empirical data. In the fMRI experiment, VS reactivity reflects the difference in striatal response to blocks of trials where the selected action gets more reinforced (i.e. win blocks) *vs* blocks of trials where the selected action gets punished more (i.e. loss blocks). To simulate this in the network, we calculated Δ_Card_, which is the difference in drift rate of the Go pathway (v^G^) and No Go pathway (v^N^) for the selected action channel on that trial. Figure 4B shows Δ_Card_ on each consecutive trial for a range of network simulations with different degrees of learning on the Go pathway (α^G^). As α^G^ increases Δ_Card_ also increases in later trials, reflecting the facilitation of the selected action channel with learning. Lower levels of α^G^ result in a network with lower overall activity across trials. Thus, Δ_Card_ serves as a reliable proxy for the reactivity of striatal nuclei. We used the average Δ_Card_ across all simulated trials in an experiment, μΔ_Card_, as a proxy for the network’s overall striatal reactivity. Using this score, we then simulated the VS reactivity and payoff associations reported in Table 2 by correlating μΔ_Card_ with payoff scores for groups of simulated agents with different asymmetries in Go (α^G^) and No Go (α^N^) learning rates (Figure 4F). To ensure that learning-rate asymmetries could account for observed VS reactivity-payoff associations in a large and diverse sample of subjects, each simulated agent plotted in Figure 4F was initiated with a randomly sampled set of model parameters to simulate individual differences in response caution, exploration–exploitation policies and decision onset time (see Methods for details). Similar to the color scheme used for Figure 4B–D, low, medium and high values of α^N^ are depicted on a red-to-blue gradient, with lighter and darker hues corresponding to lower and higher values of α^G^, respectively. Indeed, despite individual differences across all other dimensions of the model, altering the asymmetry of α^G^ and α^N^ produced systematic changes at the group level, such that when α^N^ is high (blue line), higher network reactivity is associated with greater payoff scores, maximally so when α^G^ is also high (dark blue dots). However, when α^N^ is low (red line) and decisions are asymmetrically reinforced by α^G^, higher network reactivity is associated with lower overall payoff scores and thus, worse performance.

**Fig. 5:**
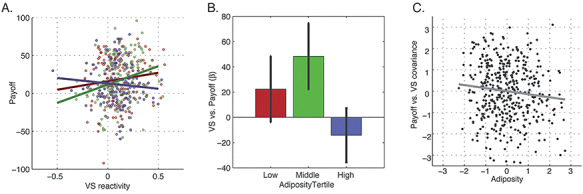
VS reactivity *vs* payoff score. (A) Scatter plots of the joint distribution between VS reactivity and payoff scores for participants in the lowest (red), middle (green) and highest (blue) tertile of the adiposity score distributions. (B) Means and 95% confidence intervals of the regression models shown in (A). (C) Joint distribution of the covariance score of payoff *vs* VS reactivity and adiposity value (see text for details).

To look for this signature in the human data, we first regressed both VS reactivity and adiposity against payoff score. While controlling for both age and gender, we observed both significant main effect of VS reactivity (${\beta}_{\mathrm{VS}}=19.181,\kern0.5em \mathrm{STD}\Big({\beta}_{\mathrm{VS}}\Big)=7.189,\kern0.5em P=0.004\Big)$ and a modest effect for adiposity (${\beta}_{\mathrm{Adiposity}}=-2.632,\kern0.5em \mathrm{STD}\Big({\beta}_{\mathrm{Adiposity}}\Big)=1.620,P=0.047\Big)$ on payoff score, as well as a VS reactivity and adiposity interaction (${\beta}_{\mathrm{VS}\times \mathrm{Adiposity}}=-15.883,\kern0.5em \mathrm{STD}\Big({\beta}_{\mathrm{VS}\times \mathrm{Adiposity}}\Big)=6.842,P=0.009\Big)$. To illustrate this interaction, we next performed a tertile split on the adiposity score and categorized subjects as having low (*N* = 153), medium (*N* = 142) or high (*N* = 138) adiposity and the payoff-VS reactivity association was measured separately for each group (Figure 5A). As predicted, after controlling for age and gender, the low (*β*_low_ = 28.818, STD(*β*_low_) = 13.719, *P* = 0.022) and medium (*β*_medium_ = 44.601, STD(*β*_medium_) = 12.791, *P* = 0.001) adiposity groups had positive directions in their associations between VS reactivity and payoff whereas the high adiposity group had a negative association trend (*β*_high_ = −14.945, STD(*β*_high_) = 10.583, *P* = 0.079). Only the low and medium groups had a statistically significant association, however, our main focus here relies on the direction of the associations themselves. Figure 5B shows the mean and 95% confidence intervals for each model shown in Figure 4A. The mean of the high adiposity group falls well outside the lower bounds of both the low and medium adiposity groups, highlighting a significant negative shift in the payoff-VS reactivity association with higher adiposity levels.

To assess the differential association between VS reactivity and payoff score by level of adiposity at the individual level, we created a payoff-VS reactivity covariance score for each subject. This was done by converting the payoff and VS scores into standard normal distributions and subtracting them. Negative covariance scores reflect individuals who are on the higher end of the payoff distribution (i.e. low impulsivity) but lower end of the VS reactivity scores or vice versa. This is consistent with an asymmetry in learning rates via low learning on negative feedback signals. Positive covariance scores, on the other hand, indicate participants who are in either the high or the low end of both the payoff score and VS reactivity distributions and reflect more symmetrical learning rates. As expected, this covariance score was negatively associated with adiposity *(β*_cov_ = −0.122, STD(*β*_cov_) = 0.061, *P* = 0.033; Figure 5C), while controlling for age and gender, meaning that a negative relationship between payoff score and VS reactivity predicted greater adiposity across individuals.

## Discussion

Here we found that the sensitivity of ventral striatal pathways to reward signals (i.e. monetary wins *vs* losses) moderates a relationship between value-based decision-making and adiposity, the latter being a precursor to obesity and poor health. Using a dynamic decision model with reinforcement learning, we show the impact that reward reactivity may have on adaptive decisions depends on the relative symmetry in how positive and negative feedback are used to update future decisions: that is, as reward reactivity increases, greater sensitivity to gains over losses leads to more strictly greedy decision policies that minimize long-term gains. Indeed, individual differences in adiposity were correlated with signatures of asymmetric learning rates in the empirical data. These results suggest that sensitivity to feedback may characterize aspects of unhealthy decisions when learned associations are disproportionately driven by positive, rather than negative, outcomes.

This pattern of learning rate asymmetries on decision-making is largely consistent with work on adaptivity in basal ganglia mediated decisions. For example, dopamine release in the VS is associated with a modulation of cost-benefit estimation on future decisions (Nasrallah *et al.,* 2011) and phasic stimulation of D2 receptors in this region of the striatum have been shown to reduce risk seeking behavior in rodents (Zalocusky *et al.,* 2016). These D2 sensitive neurons are predominantly expressed in indirect pathway cells (Cazorla *et al.,* 2014; Keeler *et al.,* 2014), suggesting that low sensitivity of indirect pathway systems may increase impulsive or risky decisions. Positron emission tomography and genetic analyses have found that individuals with greater levels of adiposity have lower D2 receptor binding than leaner counterparts (Stice *et al.,* 2008; Volkow *et al.,* 2008b; de Weijer *et al.,* 2011). This is largely consistent with the present findings; specifically, reduced phasic D2 in the VS should down regulate sensitivity to negative outcomes and thus make strictly greedy decisions that limit the ability to maximize long-term rewards. This implies that directly linking D2 pathways and asymmetric learning dynamics to impulsive decisions in the etiology of adiposity and risk for obesity should be the focus of future work.

While the present findings do provide clear evidence that adiposity may coincide with signatures of asymmetric feedback learning during value-based decision-making, it is worth noting aspects of the experimental design that may limit generalization of these findings. First, the sample consisted of predominantly mid-life, Caucasian adults. Given evidence of changes in reward learning across development (e.g. Geier *et al.,* 2010), it is likely that the degree to which effective value-based decision-making drives health behaviors varies over time. In addition, individual differences in sociocultural experiences may likely have a substantial impact on this relationship between reward learning and health behaviors (Hanson *et al.,* 2016). Second, this study took advantage of an archival sample. Predicting individual differences in off-line measures of IGT performance based on VS responses from an independent task is a coarse method for evaluating our primary hypothesis. Follow up work should use more targeted reinforcement learning tasks that allow for measuring trial-wise responses in the MRI environment that will allow for directly measuring the magnitude of learning to gains and losses, such as the multi-armed bandit task (Sutton and Barto, 1998). Finally, interpretations of our latent variable of adiposity are also limited insofar as it does not reflect the specific pathogenic contributions of visceral adiposity (e.g. as assessed by dual-energy X-ray absorptiometry). Visceral adiposity may have varied across individuals in this sample, and it is possible that it may differentially relate to reward based decision-making processes that impact health behaviors (see Shuster *et al.,* 2012 for review). These limitations do not necessarily negate the conclusions drawn from the current analysis, but point to future experimental directions to explore.

## Funding

This work was supported by National Institutes of Health grant PO1 HL040962 and a National Science Foundation CAREER Award 1351748 and by National Heart, Lung, and Blood Institute of the National Institutes of Health under Award Numbers P01HL040962.

## Declaration of interest

The authors declare no conflict of interest.
